# Supplementary material for: HIVprotI: an integrated web based platform for prediction and design of HIV proteins inhibitors
Source: J Cheminform. 2018 Mar 9;10:12. doi: 10.1186/s13321-018-0266-y (PMC5845081; doi:10.1186/s13321-018-0266-y)
Supplement: Supplementary file 2 — Additional file 2. Source code of HIVProtI web server. [file 13321_2018_266_MOESM2_ESM.zip › HIVprotI_Source-code/hivprotI/weka-3-6-6/doc/weka/gui/beans/StructureProducer.html]

StructureProducer


---


|  |  |  |
| --- | --- | --- |
| **Overview** \| **Package** \| **Class** \| **Tree** \| **Deprecated** \| **Index** \| **Help** \| **Weka's home** | |  |
| **PREV CLASS**   **NEXT CLASS** | **FRAMES**    **NO FRAMES**     **All Classes** |  |
| SUMMARY: NESTED \| FIELD \| CONSTR \| METHOD | DETAIL: FIELD \| CONSTR \| METHOD |  |


---


## weka.gui.beans Interface StructureProducer

**All Known Implementing Classes:**: ClassAssigner, ClassValuePicker, Loader

---

``` public interface StructureProducer ```

Interface for something that can describe the structure of what
is encapsulated in a named event type as an empty set of
Instances (i.e. attribute information only). For example,
batchAssociationRulesEvent can be described as LHS (String), RHS (String)
and a number of metrics (Numeric); dataSetEvent, instanceEvent,
trainingSetEvent etc. are all straightforward, since they already
encapsulate instances; textEvent can be described in terms of
the title (String) and the body (String) of the text. Sometimes
it is not possible to know the structure of the encapsulated output
in advance (e.g. certain filters may not know how many attributes
will be produced until the input data arrives), in this case
any implementing class can return null to indicate this.

**Version:**
:   $Revision: 7439 $

**Author:**
:   Mark Hall (mhall{[at]}pentaho{[dot]}com)

---

| **Method Summary** | |
| --- | --- |
| `Instances` | `getStructure(java.lang.String eventName)`             Get the structure of the output encapsulated in the named event. |

**Method Detail**

### getStructure

```
Instances getStructure(java.lang.String eventName)
```

:   Get the structure of the output encapsulated in the named
    event. If the structure can't be determined in advance of
    seeing input, or this StructureProducer does not generate
    the named event, null should be returned.

    :   **Parameters:**: `eventName` - the name of the output event that encapsulates the requested output. **Returns:**: the structure of the output encapsulated in the named event or null if it can't be determined in advance of seeing input or the named event is not generated by this StructureProduce.


---


|  |  |  |
| --- | --- | --- |
| **Overview** \| **Package** \| **Class** \| **Tree** \| **Deprecated** \| **Index** \| **Help** \| **Weka's home** | |  |
| **PREV CLASS**   **NEXT CLASS** | **FRAMES**    **NO FRAMES**     **All Classes** |  |
| SUMMARY: NESTED \| FIELD \| CONSTR \| METHOD | DETAIL: FIELD \| CONSTR \| METHOD |  |


---
